# Video gamers demonstrate superior bronchoscopy skills among beginners

**DOI:** 10.1038/s41598-024-52730-z

**Published:** 2024-01-27

**Authors:** Masafumi Shimoda, Yoshiaki Tanaka, Kozo Morimoto, Kozo Yoshimori, Ken Ohta

**Affiliations:** Respiratory Disease Center, Fukujuji Hospital, Japan Anti-Tuberculosis Association (JATA), 3-1-24 Mastuyama, Kiyose, Tokyo 204-8522 Japan

**Keywords:** Medical research, Diagnosis

## Abstract

While previous research has explored the connection between video gaming and medical procedures, studies on the connection between video gaming and bronchoscopy techniques are lacking. This study aimed to investigate how video gaming experience influences bronchoscopy skills, particularly among beginners. This study was conducted at Fukujuji Hospital from January 2021 to October 2023. Twenty-three participants were assigned to the inexperienced group, and eighteen participants were assigned to the experienced group. The observational time during bronchoscopy, measured using a simulator, and the playing time of SPLATOON 2 (NINTENDO Co. Ltd., Japan) were analyzed. Video gaming skills were assessed based on game completion time, with shorter times indicating faster task completion. Participants were also divided into gamer and nongamer subgroups for further comparisons. A moderate linear relationship existed between bronchoscopic observation time and game completion time in the inexperienced group (r = 0.453, *p* = 0.030). However, no correlation was found in the experienced group (r = 0.268, *p* = 0.283). Among the inexperienced group, the gamer subgroup (n = 12) exhibited significantly shorter bronchoscopic observation times than did the nongamer subgroup (n = 11) (median [range]: 200 [129–229] s) vs. 281 [184–342] s, *p* = 0.005). This study demonstrated a relationship between bronchoscopy technique and video gaming skills among individuals with little bronchoscopy experience.

## Introduction

Flexible bronchoscopy is a widely used procedure for the assessment, diagnosis, and treatment of patients with respiratory disease^1^. Improving bronchoscopy techniques benefits patients by reducing discomfort during bronchoscopy^2,3^. Simulation-based training for bronchoscopy has shown significant advantages in enhancing skills and behaviors^4^. Recently, bronchoscopy simulators incorporating virtual reality technology and robotic-assisted bronchoscopy, in which instruments are controlled using a handheld controller while viewing a monitor, have increased in popularity^5,6^. These advanced devices have a similar setup to that of video games. Bronchoscopic procedures require skill sets that overlap with those of video games, including visuospatial awareness, rapid decision-making, and psychomotor skills^7^. Previous reports have shown that training with video games can improve various medical procedures, such as gastrointestinal endoscopy, laparoscopic surgery, and surgical techniques^8–13^. Studies have also reported a relationship between bronchoscopy techniques and previous video game experience among beginners^7^. However, in previous reports, active video game players were defined based on their self-reports, and their gaming skills were not evaluated^7–13^. Research evaluating both bronchoscopy techniques and video gaming skills is lacking. Therefore, this study aimed to investigate how video game experience affects bronchoscopy techniques, with a specific focus on these skills among beginners.

## Materials and methods

### Study design and setting

This study was conducted prospectively at Fukujuji Hospital from January 2021 to October 2023. Two groups of participants were recruited: the inexperienced group (consisting of medical students or residents without any surgical training or experience and no prior exposure to bronchoscopy) and the experienced group (consisting of respiratory physicians who had performed bronchoscopy ≥ 100 times). The exclusion criteria were as follows: patients who underwent bronchoscopy between 1 and 99 times and those who had played the game SPLATOON 2 (NINTENDO SWITCH) without gyro aiming. One participant was excluded based on the criteria. A total of 23 participants were included in the inexperienced group; these participants consisted of ten medical students, seven junior medical residents, and six senior medical residents. Two of the 23 participants in the inexperienced group aspired to become respiratory physicians, but many participants had not yet decided on their specialty. The experienced group included 18 respiratory physicians. Data on bronchoscopy techniques, video gaming skills, and other relevant variables were collected and compared between the inexperienced and experienced groups. Additionally, participants were further divided into two subgroups: the gamer subgroup and the non-gamer subgroup. Within each subgroup, data pertaining to bronchoscopy techniques and video gaming skills were compared, both in the inexperienced group and the experienced group. This study was approved by the Institutional Review Board of Fukujuji Hospital, and all participants provided written informed consent. The decisions made by this board were in accordance with the Declaration of Helsinki (Study number: 22033, IRB approval dates: January 1st, 2023).

### Evaluation of bronchoscopy techniques

The participants’ bronchoscopy technique results were evaluated by assessing the duration of observation of the bronchial lumen (hereafter, observation time). Participants utilized a flexible bronchoscope (BF-P290, OLYMPUS Co., Ltd., Japan) on a simulator (11117-000, KYOTO KAGAKU Co., Ltd., Japan) to perform the observations in a specific order. The bronchoscope was inserted through the mouth of the simulator, and sequentially observed and capturedimages of the tracheal bifurcation, right main bronchus, right upper lobe bronchus, intermediate bronchial trunk, middle lobe branch, right S6 bronchus, right S7 bronchus, right S8-10 bronchi, tracheal bifurcation, left main bronchus, left upper lobe branch, left upper division branch, lingular branch, left lower lobe branch, left S6 bronchus, left S8-10 bronchi, and finally the tracheal bifurcation again. The inexperienced group received guidance and underwent training for approximately 10 min before the simulated bronchoscopy, and the route was guided during observation of the bronchial lumen under the supervision of an experienced doctor. The experienced group underwent bronchoscopy as usual, without any specific training or guidance.

### Evaluation of video gaming skills

The participants’ video gaming skills were assessed by having them play the game SPLATOON 2 on a Nintendo Switch console, with shorter game completion time indicating the ability to complete tasks rapidly. SPLATOON 2 is a third-person shooter game in which the objective is to paint a field with paint bullets in a team game setting. Players control a character and use a gun with paint bullets to attack enemies and paint fields. In this study, participants played a trial mode involving defeating 8 enemies. The latency to attack all the enemies was measured (hereafter, the game completion time). Prior to the evaluation, participants were given an opportunity to practice by defeating all the enemies once. All participants used the starting weapon provided in the game. The game typically utilizes gyro aiming, which involves aiming to tilt the controller, which is equipped with a gyroscope. However, participants in this study played the game with the gyro aiming turned off and used a regular controller instead. SPLATOON 2 can choose gyro aiming or joystick, which are features that were recently developed; however, players generally play video gaming by using joystick. Therefore, participants in our study used a joystick when playing SPLATOON 2. The reasons for choosing SPLATOON 2 for an evaluation of video gaming skills were as follows: a third-person shooter game, easy operation even for gaming beginners (player uses 2 levers and 1 bottom only), and the presence of an easy stage for an evaluation of video gaming skills.

### Definition of the gamer subgroup

In this study, participants were classified into two subgroups based on their video game experience. The gamer subgroup included participants with experience playing video games that involved controlling a character from a first-person or third-person perspective, such as shooter games and 3D-action games. These participants reported playing games for at least 1 h per week, and they had engaged in video gaming for 6 years or more. The definition was established according to a modified version of a previous report. The original report did not specify the duration of gaming experience; therefore, we decided to define the duration as 6 years or more, aligning with typical educational periods such as elementary school, between junior high school and high school, or university. In contrast, the non-gamer subgroup consisted of participants who either lacked prior gaming experience or who played games mainly within the genres of role-playing games, adventure games, puzzle games, and/or simulation games. These participants had limited exposure to games involving character control from a first-person or third-person perspective.

### Statistical methods

All the data were analyzed and processed using EZR, version 1.53^14^. Spearman’s correlation analysis was performed to determine the relationships among the different variables. The interpretation of Spearman’s correlation was based on the Dancey & Reidy criteria as follows: perfect (r = 1 or − 1), strong (1 > r ≥ 0.7 or − 1 > r ≥ − 0.7), moderate (0.7 > r ≥ 0.4 or − 0.7 > r ≥ − 0.4), weak (0.4 > r ≥ 0.1 or − 0.4 > r ≥ − 0.1), or zero (r = 0)^15^. The Mann–Whitney U test and Fisher’s exact test were used for group comparisons. The level of statistical significance was set at *p* = 0.05 (2-tailed).

### Ethics approval and consent to participate

The study was approved by the Institutional Review Board of Fukujuji Hospital (Study number: 22033, IRB approval dates: January 1st, 2023), and all participants provided written informed consent. The decisions made by this board are based on and in accordance with the Declaration of Helsinki.

## Results

The baseline characteristics of the study participants are summarized in Table 1. Among participants in the inexperienced group, the median age was 27 years (range: 22–36), and 15 (65.2%) were male. The median bronchoscopic observation time and the median game completion time of this group were 215 s (range: 129–342) and 45 s (range: 22–153), respectively. Among participants in the experienced group, the median age was 33 years (range: 28–49), and 15 (83.3%) were male. The median bronchoscopic observation time in the experienced group (103 s, range: 79–165 s) was significantly shorter than that in the inexperienced group (*p* < 0.001). The median game completion time in the experienced group was 41 s (range: 26–134), which was not significantly different from that in the inexperienced group (*p* = 0.478). The number of participants categorized as gamers in each group did not significantly differ, with 12 participants (52.2%) in the inexperienced group and 10 participants (55.6%) in the experienced group categorized as gamers (*p* = 1.000).

**Table 1:** Baseline characteristics of the study participants.

	Inexperienced group (n = 23)	Experienced group (n = 18)	*p* value
Age, median (range), years	27 (22–36)	33 (28–49)	< 0.001
Sex (male/female), n	15/8	15/3	0.291
Bronchoscopic observation time, median (range), s	215 (129–342)	103 (79–165)	< 0.001
Game completion time, median (range), s	45 (22–153)	41 (26–134)	0.478
Gamer, n (%)	12 (52.2)	10 (55.6)	1.000

**Figure 1:**
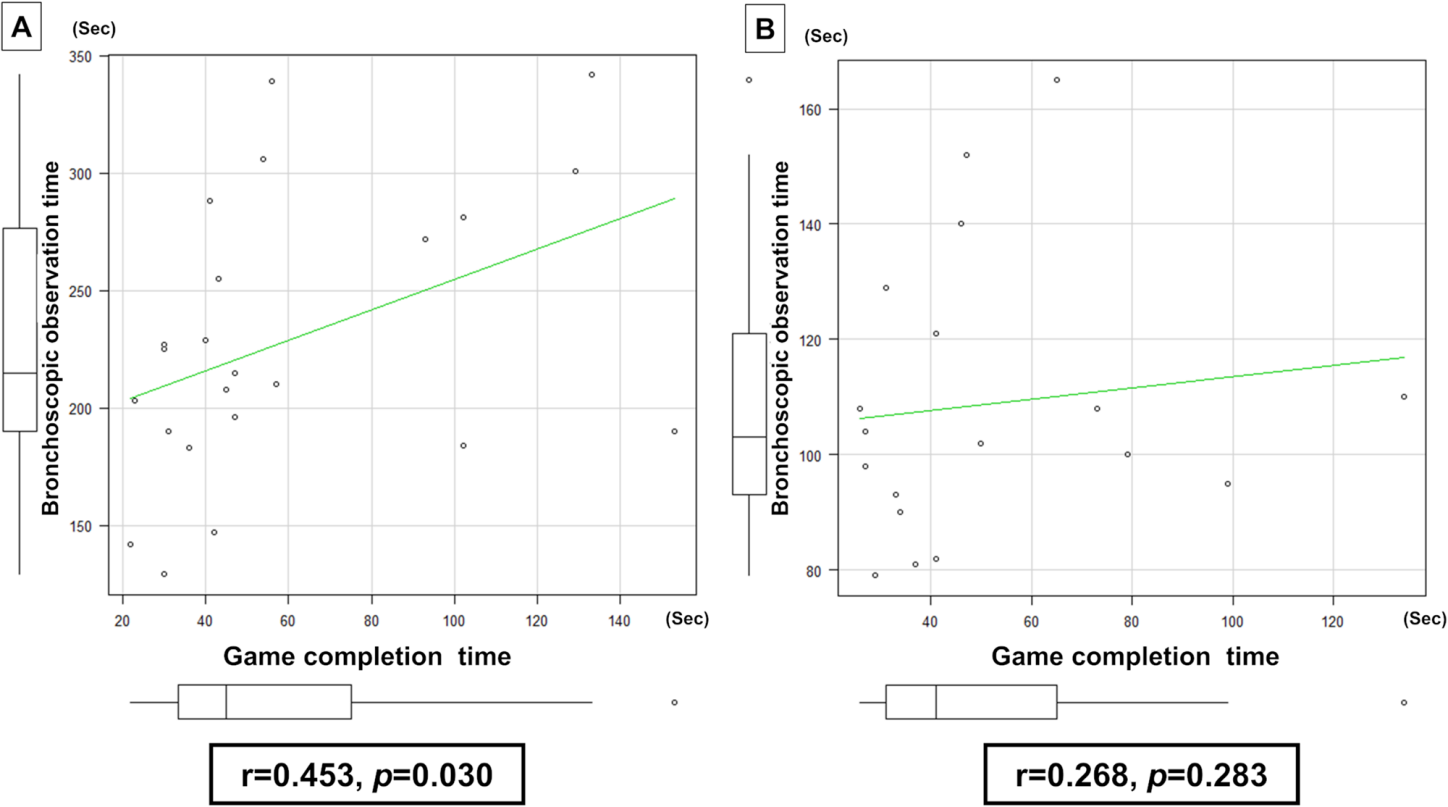
Pearson’s correlation analysis of the relationship between bronchoscopic observation time and game completion time among participants. (**A**) Within the inexperienced group, there was a moderate linear relationship between bronchoscopic observation time and game completion time (r = 0.453, *p* = 0.030). (**B**) Within the experienced group, no correlation was found between bronchoscopic observation time and game completion time (r = 0.268, *p* = 0.283).

The bronchoscopic observation time had a moderate linear relationship with the game completion time among participants in the inexperienced group (r = 0.453, *p* = 0.030) (Fig. 1A). There was no correlation between bronchoscopic observation time and game completion time among participants in the experienced group (r = 0.268, *p* = 0.283) (Fig. 1B).

**Figure 2:**
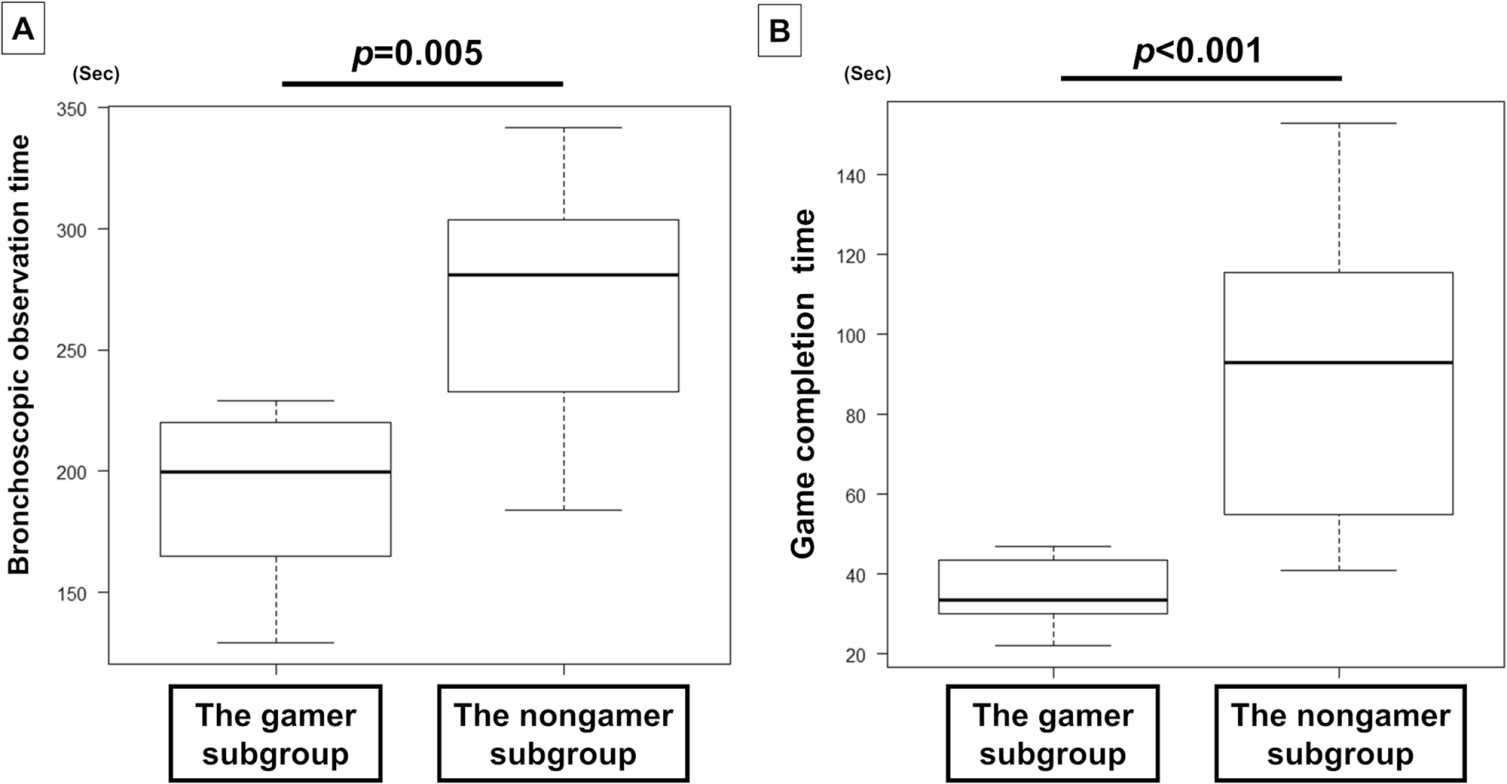
Comparison of bronchoscopic observation time and game completion time between the gamer subgroup (n = 12) and nongamer subgroup (n = 11) within the inexperienced group. (**A**) The bronchoscopic observation time in the gamer subgroup was significantly shorter than that in the nongamer subgroup (median [range]: 200 [129–229] s vs. 281 [184–342] s, *p* = 0.005). (**B**) The game completion time was shorter in the gamer subgroup than in the nongamer subgroup (median [range]: 34 [22–47] s vs. 93 [41–153] s, *p* < 0.001).

In the inexperienced group, 12 participants were in the gamer subgroup, and 11 participants were in the nongamer subgroup. The bronchoscopic observation time in the gamer subgroup was significantly shorter than that in the nongamer subgroup within the inexperienced group (median [range]: 200 [129–229] s vs. 281 [184–342] s, *p* = 0.005) (Fig. 2A). The game completion time was also shorter in the gamer subgroup than in the nongamer subgroup within the inexperienced group (median [range]: 34 [22–47] s vs. 93 [41–153] s, *p* < 0.001) (Fig. 2B). In the experienced group, there were 10 participants in the gamer subgroup and 8 participants in the nongamer subgroup. There was no significant difference in the bronchoscopic observation time between the gamer subgroup and the nongamer subgroup within the experienced group (median [range]: 101 [79–152] s vs. 105 [82–165] s, *p* = 0.534). However, the game completion time in the gamer subgroup was significantly shorter than that in the nongamer subgroup within the experienced group (median [range]: 34 [26–47] s vs. 69 [31–134] s, *p* = 0.006).

## Discussion

This study examined the relationship between bronchoscopy techniques and video game playing skills. Within the inexperienced group, there was a correlation between bronchoscopic observation time and game completion time, indicating that participants with better video game skills were faster at performing bronchoscopic observation. Presumably, individuals who were accustomed to gaming may exhibit better baseline handling of bronchoscopy techniques, as suggested by a previous report^7^. However, a previous report showed that an 8-week video gaming program involving 2.5 h of training every week did not lead to mastery of bronchoscopy techniques^7^. One of the gamers’ skills is related to the use of handheld controllers for character manipulation on screens. Gamers have developed a sense that their controller-based manual actions correlate with on-screen actions, and this skill enables gamers to excel in playing even new games more proficiently. Gamers acquire skills through prolonged gaming habits. We believe that this skill could help gamers intuitively understand how to perform bronchoscopy and expedite their mastery of bronchoscopy techniques. Conversely, in the experienced group, there was no significant difference in bronchoscopic observation time according to gaming experience. This finding suggested that, in practice, operators can acquire proficiency in bronchoscopy regardless of their video gaming experience. Patients often experience discomfort during bronchoscopy^16^, and skilled operators can help reduce patient discomfort during bronchoscopy^2,3^, emphasizing the importance of acquiring proficient bronchoscopy techniques^4^.

Bronchoscopic procedures share similarities with video gaming^7^, as operators navigate through the bronchi displayed on a monitor and manipulate devices to perform tasks. Bronchoscopy is performed from a first-person perspective and with devices (such as forceps) aimed at accessing and inserted into the targeted bronchi. This system appears to involve skills similar to those of playing 3D shooter games, such as first-person or third-person shooter games. A previous report also indicated a lower airway collision rate during bronchoscopy among the gamer group^7^, while our study did not investigate bronchoscopy techniques based on assessment tools such as the Bronchoscopic Skills and Tasks Assessment Tool and the Bronchoscopy Step-by-Step Evaluation Tool, which is a validated tool for assessing competency in bronchoscopy^17^. In addition to bronchoscopy, previous studies have reported medical procedures. A video gaming experience provides benefits in laparoscopy^11,18,19^, robotic surgery^11,20,21^, gastrointestinal endoscopy^22^, arthroscopy^23^, ophthalmic surgery^24^, and endotracheal intubation^25^, and eye-hand and visual-spatial skills training through video gaming could be associated with various medical procedures. Therefore, gamers who are accustomed to using controllers to shift their position as indicated on a monitor may excel in performing bronchoscopy due to their gaming experience. Additionally, high visuospatial awareness, which is enhanced through gaming, may also enhance bronchoscopy techniques^7^. Video gaming has been shown to improve the ability to learn new tasks^26^ and enhance cortical plasticity and performance of minimally invasive skills^8,27^. While operators can improve their bronchoscopy techniques over time^4,5^, gamers may require less practice to achieve proficiency. Prolonged examination time during bronchoscopy can lead to intense coughing^2,16^, highlighting the benefits of operators who are proficient with advanced techniques that minimize the duration of the procedure^2,3^. Furthermore, simulation-based bronchoscopy training, including virtual reality gaming aspects, has also attracted attention for leading to improved skills in recent years^5^; thus, gaming experiences may further benefit beginners. However, the actual utilization of these training devices may not be high, potentially due to a lack of simulator availability^28^. It is considered that the application of games to education will become more important. On the other hand, brief exposure to video gaming has been found to have no impact on bronchoscopic procedures^7^. Therefore, tailored learning programs should be developed for individual learners, and considering their gaming experience and skills might prove beneficial.

Importantly, video gaming might have both positive and negative effects. While there are concerns about the potential adverse effects of video gaming on children, such as increased aggression, decreased empathy, or decreased academic performance^29^, the influences of gaming are generally minimal^30–32^. Furthermore, gaming during puberty has been associated with a temporary psychological state called Chūnibyō, in which children exhibit media-inspired behaviors, in Japan^33^. However, importantly, Chūnibyō is not considered a disease, and we do not believe that gaming has negative effects on children during puberty^33^. Studies have demonstrated the benefits of gaming for improving visuomotor control^27,34^, cascading action^35^, and information processing^36^ as well as increasing motivation through reward mechanisms^7,37^. It is crucial to prevent excessive reliance on gaming^38^; however, appropriate gaming experiences and training may have transferable learning benefits beyond video games^26^. Overall, video gaming has potential applications in the field of medicine as techniques continue to improve.

This investigation has several limitations. The study was conducted at a single clinical center. The sample size was small, although a significant relationship was observed between bronchoscopic observation time and video gaming time in the inexperienced group. Based on the sample size calculation for the comparison of mean values between two groups conducted in advance, each group (gamer and non-gamer groups) in the inexperienced group needed 9 participants. In the experienced group, each group needed 7 participants based on a non-inferior sample size calculation for the comparison of mean values between the two groups. Two participants had prior experience playing SPLATOON 2 before this study; however, they played with gyro aiming. All participants in this study used a joystick to play SPLATOON 2, which differs from gyro aiming. Therefore, their previous experience with gyro aiming at SPLATOON 2 may not have affected the recorded game completion time. We choose SPLATOON 2 for an evaluation of video gaming skills because of the use of a third-person shooter game, easy operation, and easy stage. A first-person shooting game might be more suitable for assessing bronchoscopic technique efficacy. However, most first-person shooting games feature too complex operations and stages for beginners, which may not be useful for effectively assessing participants’ gaming skills. Both first-person and third-person shooter games require aiming and shooting at targets and share many common controls. Therefore, we believe that the bias introduced by this difference is minimal. Another limitation is that only bronchoscopic observation time was used to evaluate bronchoscopy technique, and other factors, such as airway collision rates and the Purdue Visual Spatial test, were not evaluated. Generally, beginners focus on learning to perform bronchoscopic observation, and this technique becomes the basis of further techniques, such as bronchoalveolar lavage, brushing, and forceps biopsy; therefore, we chose to assess bronchoscopic observation time in this study. The specialty of participants in the inexperienced group and their aspiration of airway anatomy knowledge may have differed among participants in the inexperienced group. This may bias the results of this study.

## Conclusion

This study demonstrated a correlation between bronchoscopy technique and video gaming skills among beginners lacking experience performing bronchoscopy. This finding suggested that individuals who are accustomed to playing video games may have rapid access to bronchoscopy techniques.

## Data Availability

The datasets used and/or analyzed during the current study are available from the corresponding author upon reasonable request.
